# An operational distinction between quantum entanglement and classical non-separability

**DOI:** 10.1098/rsta.2023.0342

**Published:** 2024-12-24

**Authors:** Natalia Korolkova, Luis Sánchez-Soto, Gerd Leuchs

**Affiliations:** ^1^ School of Physics and Astronomy, University of St. Andrews, North Haugh, St. Andrews, Fife KY16 9SS, UK; ^2^ Max-Planck-Institut für die Physik des Lichts, Staudtstraße 2, Erlangen 91058, Germany; ^3^ Departamento de Óptica, Facultad de Física, Universidad Complutense, Madrid 28040, Spain; ^4^ Institut für Optik, Information und Photonik, Universität Erlangen-Nürnberg, Erlangen 91058, Germany; ^5^ Department of Physics, University of Ottawa, Ottawa, Ontario K1N6N5, Canada

**Keywords:** quantum entanglement, classical entanglement, quantum measurement, non-separability

## Abstract

Quantum entanglement describes superposition states in multi-dimensional systems—at least two partite—which cannot be factorized and are thus non-separable. Non-separable states also exist in classical theories involving vector spaces. In both cases, it is possible to violate a Bell-like inequality. This has led to controversial discussions, which we resolve by identifying an operational distinction between the classical and quantum cases.

This article is part of the theme issue ‘The quantum theory of light’.

## 1. Introduction

In 1997, Spreeuw first discussed ‘a classical analogy of entanglement’ [1], followed 4 years later by a paper elaborating on the classical analogy of quantum information processing using wave optics [2]. Depriving quantum entanglement of its purely quantum nature and exploring the new capabilities of classical optics have sparked a range of intriguing theoretical considerations and experiments (for reviews, see [3–6]).

This exploration has prompted a controversial discussion, the essence of which we highlight with a few selected examples. In 2011, Qian and Eberly suggested a new interpretation of light polarization based on ‘non-quantum entanglement’, stating that ‘polarization is a characterization of the correlation between the vector nature and the statistical nature of the light field’. Subsequent elaborations on the emerging links between quantum and classical optics [7,8] led Eberly *et al*. to a strong statement: ... ‘entanglement is a vector space property, present in any theory with a vector space framework. There is no distinction between quantum and classical entanglements, as such’ [8]. This assertion has elicited equally strong opposing statements. As has been highlighted in discussions on this topic in *Science* [9], ‘Entanglement is a property of the quantum world; classical systems need not apply’. Karimi & Boyd wrote, referring to the ‘classical entanglement’ term: ‘We do not endorse this new nomenclature,’ adding, ‘... this situation lacks the key feature - nonlocality’ [9].

Despite the wide range of opinions, the past decade has witnessed a plethora of potentially useful experiments with vector optics and other classical implementations of vector spaces, demonstrating the power of ‘classical entanglement’ (or rather non-separability) in high-precision measurements, metrology applications and in emulation of various quantum information protocols. However, the controversy in interpreting these experiments and discussions about the fundamental nature of entanglement, both quantum and classical, remains ongoing [6].

We address the controversy by recognizing that the term should rely solely on the details of the experimental observation rather than any prior knowledge about the system. As we will explain, concepts like non-locality or counting the number of particles involved fail to offer a clear distinction between quantum and classical entanglement.

Instead, there is a decisive difference: the characterization of quantum entanglement always involves correlating statistical outcomes of multiple measurements on a given system, as elaborated below. Conversely, classical entanglement typically refers to deterministic correlations between a single measurement and a filter or sorter operation, such as those imposed on a light beam by a polarizing beam splitter or another quantum sorter [10,11]. The settings of such filters or sorters do not involve measurement (and is a unitary operation). This provides a clear distinction between the two phenomena.

## 2. Entanglement revisited: questions to ask

The term entanglement refers to a unique form of correlation between two or more different variables (for an excellent recent discussion of quantum entanglement, see [12]). Mathematically, entangled states are described by non-separable functions within the vector space known as Hilbert space. Their global nature leads to the puzzling property that the outcome of a measurement on one part of such a non-separable state at one location appears to have an instantaneous effect on the other part of the state at another location, resulting in perfect correlations between measurement results at both locations. However, this ‘immediate action’ cannot be detected solely by measuring at one location.

The emergence of the ‘non-local effect’ from a local measurement stems from quantum theory, giving rise to several conceptual questions. Opponents of this difficult-to-grasp action at a distance brought up explanations rooted in classical physics. This was used by John Bell to demonstrate that quantum physics cannot be explained by classical stochastic theories relying on local hidden variables, hence coining the term quantum entanglement [13].

The non-local nature of correlations in quantum entanglement stems from non-separability. However, similar types of correlations can be found in a classical description involving mathematically non-separable functions. Are we discussing here correlations of a common nature, as suggested, for example, by Eberly *et al*? The term ‘classical entanglement’ was coined. Is such terminology appropriate? Does any mathematical non-separability imply Bell-type non-locality, and is it this non-locality that makes up entanglement?

John Bell emphasized that his analysis applies to situations involving two distinctly different particles. This establishes a clear and naturally unique partitioning of the Hilbert space of the composite quantum system, as well as the measurements to be performed. However, the choice of partitioning becomes less obvious when different degrees of freedom contribute to the dimensions of the Hilbert space, particularly in discussions involving light and mode functions. Extending these discussions to such scenarios has recently gained attention, partly in relation to the debate on quantum versus classical entanglement.

In a recent study by Paneru *et al*. [12], quantum contextuality has been used as a tool to identify the genuine quantum nature of correlations between different degrees of freedom. Realism, locality and non-contextuality for single and multiple particles represent different aspects of physical theories. Paneru *et al*. argue that classical states of light, whether separable or non-separable, can be fully described using the wave picture without invoking field quantization. Therefore, they cannot challenge these fundamental concepts in classical physics (for an introduction to field quantization and the quantum theory of light, see [14]).

**Figure 1.**
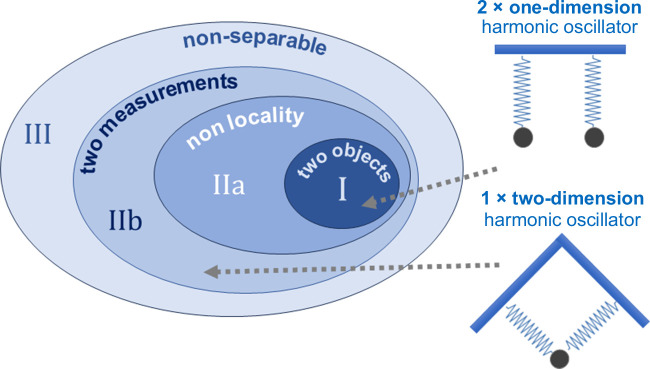
Left: different possible subsets of a set of general non-separable states. The ellipses represent the four decisive properties of systems under consideration: consist of two objects, exhibit non-locality, allow for two measurements and can be written as mathematically non-separable mode functions. The examples of physical systems populating a particular set are described in detail in the text: I - non-separable states involving two excitations (two objects); IIa,b - non-separable states with only one single excitation (object), but where two distinct measurements can still be performed on two non-separable partitions of the Hilbert space. III - classically entangled states described by non-separable mode functions. Right: two paradigmatic examples of the states of type I and IIb, both allowing for two distinct, independent measurements - see text for discussion.

We want to address the problem from a different, more hands-on viewpoint. Realism, locality and non-contextuality are all related to a measurement process. Figure 1 visually highlights some of the non-trivial aspects of partitioning and the role of measurement in analysing correlations in non-separable systems. For simplicity, we restrict ourselves to bi-partite non-separable states. Different possible partitions of the *mathematical* vector space can be of quite different *physical* nature [15]. They can correspond to objects of zero or non-zero rest mass, such as a photon or an atom, respectively, or to mode functions, such as particular spatial modes.

The crucial role of partitioning was explicitly highlighted in the early 2000s as an ambiguity and hence freedom in selecting a multi-partite structure for a given system^1^. This also renders the presence of entanglement and its degree ambiguous [16,17]. In [16], Zanardi writes: ‘Clearly even the notion of entanglement is affected by some ambiguity being relative to the selected multipartite structure...The above ambiguity is removed as soon as, according to some criterion, a preferred multipartite structure is selected among the family of all possible partitions into subsystems’. These papers hence aimed at presenting a way to control the amount of achievable entanglement via the partitioning choice. Their main body is devoted to developing a corresponding mathematical set-up, based on the ideas of virtual quantum systems and compoundness notion [16], leading to a general algebraic framework where the desired quantum tensor product structure is *observable induced* [17]. The interested reader is referred to [16,17], as the presented theory is beyond the scope of our work and not relevant to our discussion here. We share, however, one of the key realizations, stated already in these papers: ‘a partitioning of a given Hilbert space is induced by the experimentally accessible observables (interactions and measurements)’ [17]. Or putting it differently: ‘the system S is viewed as composed by $S_{1}$, $S_{2}$, . . . if one has some operational access (is able to ‘access’, ‘control’, ‘measure’) to the individual degrees of freedom of $S_{1}$, $S_{2}$’ [16].

For the task we set here, the operational distinction between quantum and classical non-separability, this crucial point should be formulated differently: it is not the partitioning choice but the physical nature of the given subsystems that is a key issue. This consideration becomes particularly profound in the realm of optics. To reiterate, the complication stems from the following observation: non-separability is a mathematical property and is independent of the physical nature of the different degrees of freedom, i.e. the different parts of Hilbert space, whereas the ability to perform one or more measurements does depend on the physical nature of the degree of freedom. A measurement requires some excitation, some energy above the level of the vacuum, which can be associated either with a zero or non-zero rest mass particle or a combination thereof. Mode functions in optics, for example, can have numerous degrees of freedom occupying a larger Hilbert space, but they are merely frames for excitations, and by themselves, they do not refer to any excitation. If the mode is not excited, there is nothing specific to measure (other than non-specific vacuum fluctuation). We, therefore, attempt to structure the types of non-separable states with regard to their physical nature. We take the liberty here to use the word ‘excitation’ in a wider sense. We speak about excitation not only when we have some non-zero energy state of the electromagnetic field, but we can also view a massive particle as a de Broglie wave packet. We can then also think of ‘modes’, which are ‘excited’ or ‘not excited’, meaning the particle is there or not. Furthermore, there is a whole range of quasi-particles, meaning a combined excitation from entities of different physical natures: phonons, excitons and polaritons, to name a few. To emphasize this aspect we are writing the wave functions also in the following form, underlining that the degrees of freedoms provide the space where excitations can live:


(2.1) $$\begin{array}{rl} \left|\Psi \right\rangle & =|excitation\rangle_{degrees\;of\;freedom} \end{array}.$$

We list below the properties defining principally different cases of non-separability and refer to excitations to accommodate massive particles, photons or ‘inter-species’ excitations.

### (a) Defining properties of non-separable systems of different types

We want to single out four defining properties that lead to non-separable states of principally different physical nature (see figure 1). We will elucidate their distinctive features with examples in the next subsection.

#### (i) Two objects

The dark-blue innermost set in figure 1 encompasses systems with two distinct objects (i.e. excitations) living in the sub-spaces of a bi-partitioned Hilbert space. These objects are in a state described by mathematically non-separable mode functions. Here, the available ‘modes’ are populated by two excitations, rendering two distinct separate measurements (each on one of the excitations) possible. Examples of such systems are two electrons in a singlet state (spin up/down, |↑⟩,|↓⟩) or two photons of orthogonal polarization (polarization horizontal/vertical, |H⟩,|V⟩) in different spatio-temporal modes. In the literature, we usually see them in the following form:


(2.2) $$\begin{array}{rl} |\Psi_{\text{singlet}}\rangle_{AB} & =\frac{1}{\sqrt{2}}\left(|\;\;↑\;\;\rangle_{A}|\;\;↓\;\;\rangle_{B}-|\;\;↓\;\;\rangle_{A}|\;\;↑\rangle_{B}\right),\\ |\Psi_{\text{pol}}\rangle_{AB} & =\frac{1}{\sqrt{2}}\left(|H\rangle_{A}|V\rangle_{B}-|V\rangle_{A}|H\rangle_{B}\right). \end{array}$$

In order to facilitate the comparison between different scenarios, we will use the notation of equation (2.1) so that the above reads in terms of ‘modes’ and excitations as follows:


(2.3) $$\begin{array}{rl} \left|\Psi_{singlet}\right\rangle & =\frac{1}{\sqrt{2}}\left(|1\rangle_{A,↑}|1\rangle_{B,↓}-|1\rangle_{A,↓}|1\rangle_{B,↑}\right),\\ \left|\Psi_{pol}\right\rangle & =\frac{1}{\sqrt{2}}\left(|1\rangle_{modeA,H}|1\rangle_{modeB,V}-|1\rangle_{modeA,V}|1\rangle_{modeB,H}\right). \end{array}$$

Here, for example, the mode index {A,↑} denotes ‘a mode’ understood as a framework for excitation at location A in the form of a particle in spin up (see equation (2.1) and discussion before it). In the corresponding Dirac ket, $|1\rangle_{A,↑}$, ‘1’ means that this mode is indeed filled with this excitation, that is, particle in spin-up state is present at location A. The ket $|0\rangle_{A,↑}$ would mean no spin-up particle at A. In the second equation, ‘a mode’ {modeA,H} is a spatial mode A framing an excitation corresponding to a photon in A, polarized horizontally, with 1 in the ket meaning ‘this mode is excited’, that is, we have a horizontally polarized photon.

The mathematically non-separable mode functions in equation (2.3) correspond to the possible combinations of outcomes of two projective measurements defined by the excitations present. This subset, depicted in figure 1 by the dark central ellipse, marks the subset that we historically perceive as *the* entanglement. Elements of this subset possess all the four properties mentioned in figure 1: (i) they consist of two distinctly different objects, (ii) two measurements are possible, i.e. each of the two subsystems into which the system is partitioned can be measured separately, (iii) they are non-local, and (iv) they are non-separable. Correlations are manifested through a series of two measurements performed on the two subsystems involved and constitute statistical correlations.

#### (ii) Non-local

The next medium-blue shaded subset includes all states that exhibit the property of non-locality (in its trivial sense: parts of a composite system are at spatially separated locations). This may require merely one photon [18]:


(2.4) $$\begin{array}{ccc} & \begin{array}{cc} |\Psi_{\mathrm{pol}}\rangle & =\frac{1}{\sqrt{2}}\left(|1\rangle_{\mathrm{modeA}}|0\rangle_{\mathrm{modeB}}-|0\rangle_{\mathrm{modeA}}|1\rangle_{\mathrm{modeB}}\right). \end{array} & \end{array}$$

We briefly discuss non-locality in more general sense in §4, but it is deliberately not the subject of this paper.

#### (iii) Two measurements

The larger ellipse of the yet lighter blue includes all states characterized by a common feature: the possibility to perform two distinct, independent projective measurements. Remarkably, two of the three subsets contained within the ellipse labelled ‘two measurements’ deprive us of the first traditional ingredient: two objects. Here, in these two lighter-shaded subsets, there is only one single excitation initially. However, importantly, it still clearly defines two Hilbert space partitions. These partitions are determined by two distinct excitations generated by one single object, examples are given in the next section (see the second line in equation (2.6)). The mathematically non-separable mode functions describe the possible combination of measurement outcomes defined by these secondary excitations.

#### (iv) Non-separable

For states in the outermost ellipse (but outside the other subsets), we now relinquish the possibility of performing two measurements. In this class, the only remaining property is mathematical non-separability, which is also common to all cases listed above. This non-separability is not tied to excitations, and may even be demonstrated with a single coherent state excitation |α⟩, for example, as discussed in [5] at the end of §4.1.: decomposition in a particular basis followed by sorting/filtering determined by this basis can yield correlations emulating those of entangled state:


(2.5) $$\begin{array}{ccc} & \begin{array}{cc} |\Psi_{non-sep}\rangle & =|\alpha \rangle_{\left(modeA,H\right)-\left(modeB,V\right)}. \end{array} & \end{array}$$

Here, the mode function characterized by spatio-temporal mode A and horizontal polarization is written as {modeA,H}. For details see [5].

### (b) Paradigmatic examples of non-separable systems

In this subsection, we aim to explore specific physical implementations of the non-separable states encompassed in different subsets in figure 1, emphasizing the features that define their entanglement properties.

#### (i) Subset I

This is an example of prototypical quantum entanglement, the most familiar among physical implementations of non-separable states. These states belong to the type depicted in figure 1, top left, and presented in equation (2.2).

#### (ii) Subset IIa

Consider a single excitation shared by two mode functions, involving non-separability. The seminal example of such quantum entanglement is the entanglement of a single photon (or atom) incident on a beam splitter. The state at the output of the beam splitter can be written as $|\psi_{12}\rangle \propto |1\rangle_{1}|0\rangle_{2}+|0\rangle_{1}|1\rangle_{2}$ (see equation 2.4), where $|1\rangle_{j}$ refers to a photon (or atom) in output j and $|0\rangle_{j}$ represents no photon (or atom), with j=1,2.

In optics, there has been a debate on whether this scenario truly represents quantum entanglement, a controversy that was resolved in 2004 [18]. This debate is relevant to our current discussion, as the argument against entanglement was based on only one excitation being present and measurable at the output with on-off single photon detectors. However, as it is well known, an optical beam splitter always has two inputs and two outputs. The incident light field excitation determines the set of modes we are looking at and, consequently, the two output modes of the beam splitter.

In the case of an ‘undivisible’ single photon, one output mode contains a single photon while the other exhibits vacuum fluctuations in the mode defined by this excitation and vice versa. Both of these secondary excitations (with one corresponding to the ground state) can be measured using homodyne detection. Thus, although only one physical excitation is present (i.e. one photon), two measurements can still be performed at the output using homodyning, providing evidence of entanglement (see also [19]). This example IIa, also features the property of non-locality (see figure 1).

#### (iii) Subset IIb

In this scenario, we consider a single massive object that undergoes further excitations in two ways, e.g. motion in two dimensions. A seminal example of this is a single two-dimensional harmonic oscillator with possible excitations in both the x- and y-directions. An intriguing observation made by Asher Peres in his textbook [20] nearly 30 years ago (and seldom cited) clarifies this example and underscores the significance of two measurements defined by two excitations in discussions about quantum entanglement. The right part of figure 1 displays the two systems Peres discusses. Two one-dimensional harmonic oscillators coupled in a non-separable way (figure 1, top right) are a generic example of quantum entanglement, ticking all four boxes as in dark blue subset. However, mathematically this system is completely equivalent to a single mass on two springs, a single two-dimensional oscillator (figure 1, bottom right) [20]. Consequently, the wave functions of the energy eigenstates are completely equivalent in both cases:


(2.6) $$\begin{array}{ccc} & \begin{array}{cc} |\Psi_{2\times 1D}\rangle_{AB} & =\frac{1}{\sqrt{2}}\left(|n\rangle_{A}|m\rangle_{B}-|m\rangle_{A}|n\rangle_{B}\right),\\ |\Psi_{1\times 2D}\rangle_{xy} & =\frac{1}{\sqrt{2}}\left(|n\rangle_{x}|m\rangle_{y}-|m\rangle_{x}|n\rangle_{y}\right). \end{array} & \end{array}$$

Here, {|n,m⟩} denotes two possible energy levels associated with two excitations, either in the two oscillators at locations A and B, for two one-dimensional systems, or with two spatial directions x and y of a two-dimensional system.

Crucially, similar to example IIa, there are two distinct secondary excitations. Hence, despite containing only one mass, this system allows for two distinct measurements to be performed, thus decisively manifesting the entanglement property^2^.

A two-dimensional oscillator provides a prototypic example of such states, but the subset is not restricted to two-dimensional systems. Non-separable states involving quantum entanglement between two different degrees of freedom exhibit the same property. A vivid example of a locally entangled state is a mesoscopic Schrödinger cat-like state of cold atoms realized with trapped Be ions in 1996 in the group of Wineland [21]. They created the following state of a single ${\;}^{9}{Be}^{+}$ laser-cooled ion:


(2.7) $$\begin{array}{ccc} & |\psi \rangle =\frac{1}{\sqrt{2}}(|x_{1}\rangle |↑\rangle +|x_{2}\rangle |↓\rangle )\;. & \end{array}$$

Here, $|x_{j}\rangle$ denotes localized wave-packet states corresponding to two spatial positions of the atom, while |↑⟩,|↓⟩ are two distinct internal electronic quantum states of the atom (hyperfine ground states). The secondary excitations present for this single atom can be modelled as a two-dimensional harmonic oscillator or even interpreted as a Schrödinger cat-like state of two spatially separated coherent harmonic oscillator states, $|x_{j}\rangle$, j=1,2 (as suggested in [21]). The key point here is that two measurements on separate excitations of two different degrees of freedom, $|x_{j}\rangle$ and |↑⟩,|↓⟩, are possible for the state (2.7). Therefore, the state (2.7) is prototypic for ‘local entanglement’ of states that cannot be separated spatially. In other words, such states not only lack the ‘two-objects’ property, but also lack another traditional feature, non-locality.

#### (iv) Subset III

All the examples discussed are characterized by mathematically non-separable functions, as shown in figure 1. Now, consider the region of the full set outside the three inner subsets. In contrast to the previous examples, here, the partition of the Hilbert space is not defined by two excitations, and is thus representing a case of classical entanglement.

For classically entangled non-separable states, the Hilbert space is spanned by the available mode functions, and a single excitation, which can, in principle, be assigned to (included in) any of the partitions. This assignment is guided solely by experimental convenience or our choice of which measurements to perform.

Let us illustrate this with an example. In a number of metrology tasks, performance enhancement has been demonstrated using a classically entangled state with correlations between the transverse spatial mode and polarization. This is the situation formally displayed in equation (2.5). Often, however, the distinction between excitation and mode functions is not carved out so explicitly, and one finds the following notation (see [4]):


(2.8) $$\begin{array}{ccc} & |\Psi \rangle \propto |\psi_{01}(𝐫)\rangle_{A}|H\rangle_{B}-|\psi_{10}(𝐫)\rangle_{A}|V\rangle_{B}. & \end{array}$$

As we mentioned at the beginning of our discussion on figure 1, quantum mechanics stems from observations, namely measurements. In the case of equation (2.8), we are dealing with the non-separability of a vector space described entirely by available mathematical mode functions connected to different degrees of freedom. The excitation enables a measurement and the non-separable mode functions as a whole form a product state. This excitation, thus, does not contribute to the entanglement.

Contrast this with all the previous cases: there, the partitioning of the Hilbert space has been based on physical excitations. The Dirac kets corresponded to the chosen bases for the respective vector sub-spaces, and their eigenvalues were the possible measurement outcomes of the two projective measurements, one on each partition.

For equation (2.8), the partitioning initially is not connected to any excitations and the eigenvalues of the basis kets are *mode-functions, possible decomposition of the initial state in some basis*. As we will discuss in detail later, one of the eigenvalues is *the conditional measurement outcome of one single projective measurement on a chosen partition*, conditioned on the deterministic choice of the value of the other variable. That is, to unlock the physical nature of the state equation (2.8) and to connect it to observations, we rather need to view this equation in the spirit of equation (2.5).

The Hilbert space has a partition 1 spanned by two mode functions k and $k^{\prime }$ corresponding to the first degree of freedom (spatial mode), and partition 2 spanned by two mode functions l and $l^{\prime }$ corresponding to the second degree of freedom (polarization mode). However, the excitation present is independent of these partitions and corresponds to the electromagnetic wave, which can be written on the basis of various modes. Hence, the physics behind equation (2.8) is better captured by rewriting it as


(2.9) $$\begin{array}{ccc} & |\Psi \rangle \propto [|\psi_{k}\rangle_{1}|\psi_{l}\rangle_{2}-|\psi_{k^{\prime }}\rangle_{1}|\psi_{l^{\prime }}\rangle_{2}]\;|E(𝐫,t)\rangle , & \end{array}$$

where E(𝐫,t) describes the excitation of the electromagnetic field. Put simply, we have decomposed the electromagnetic wave into two spatial modes: $\psi_{01}$ horizontally polarized and $\psi_{10}$ vertically polarized (cf equation 2.5). Modifying the notation in this way brings us a bit closer to equation (2.1), which clearly emphasizes what the mode (subscript) is and what is the excitation (argument of the ket). In this system, we cannot violate realism, a cornerstone of classical physics absent in quantum mechanics, Here, we are dealing with measurement outcomes predetermined before measurement, evident in the correlations observed.

**Figure 2.**
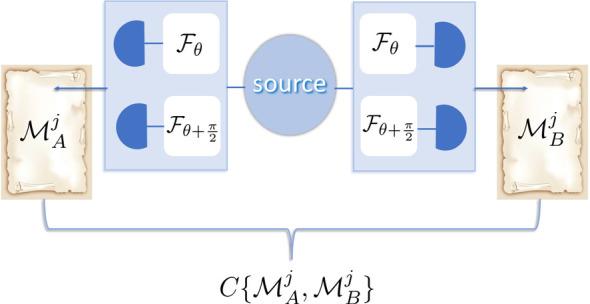
Correlations in quantum entanglement (equation 3.1). Two distinct projective measurements on subsystems A,B are performed depicted by two boxes, with a possibility of detection in two bases of choice, $F_{\theta },F_{\theta +\frac{\pi }{2}}$, and with statistical outcomes $M_{A,B}^{j}$. There are statistical correlations between measurement outcomes, $C\{M_{A}^{j},M_{B}^{j}\}$.

**Figure 3.**
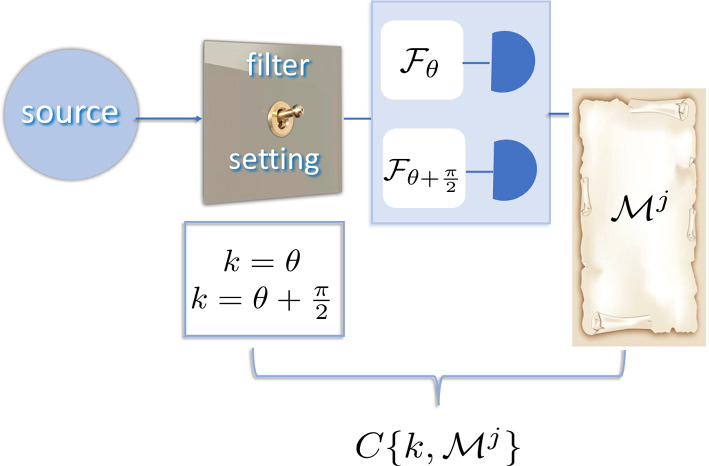
Correlations in classical entanglement (equation 3.2). Only one projective measurement described by mode function B is performed, denoted by a box with a possibility of detection in two bases of choice, $F_{\theta },F_{\theta +\frac{\pi }{2}}$ and with outcome $M^{j}$. The other mode function, A, describes a unitary operation of ’a switch', determining the filter setting k, $F_{\theta }$ or $F_{\theta +\frac{\pi }{2}}$. There is a deterministic correlation between the filter setting k and the measurement outcome, $C\{k,M^{j}\}$.

This is the key difference to the quantum entanglement of equation (2.7), which is also local but which displays two correlated excitations. Classical non-separability of equations (2.5), (2.8) and (2.9) is associated with only this one excitation. Exactly this difference is captured in figures 2 and 3—we need to compare the measurement procedures, as we elaborate in detail in the next section. The two states are deceptively similar: both of them do not possess the non-locality property and exhibit correlations between two internal degrees of freedom. Both can be written as a non-separable state in a composite vector space. However, for the quantum state of equation (2.7), the partitioning of Hilbert space is dictated by more than one excitation present, hence two independent measurements can be performed, one on each partition, and their statistics and correlation can be verified (see also [16,17] and our discussion on it in §2). As we already highlighted above, the situation is drastically different in the case of classical entanglement of equation (2.8); and this is not a question of being able to perform a certain type of measurement or not, but rather a problem of having nothing specific to measure in one of the partitions.

Note that the latter statement is also relevant to differentiate between IIa and III. For IIa, the input beam splitter modes determine a vacuum mode at one of the outputs of this beam splitter alongside a photon in the other one and vice versa. Tracing out one of the output modes, the remaining output mode will be in a mixed state excited by a fraction of a photon on average. Both these correlated output modes, each with a fractional excitation, can be measured demonstrating the correlation, provided the right measurement is done, which is homodyne detection in this case [18]. In a way, in the case of states in III, there is a free choice of where to assign the one and only available excitation |E(𝐫,t)⟩, as illustrated by equation (2.9). Formally, the excitation can be assigned to any one of the mode function partitions. Let us elaborate on what this implies for a measurement process.

To elucidate the relationship between the spatial mode and polarization, we can opt for one of the two possible scenarios. First, assume we aim to measure polarization. We assign excitation to partition 2 and conduct measurement ${\hat{M}}_{2}$ in the basis {H,V}, where potential outcomes are denoted $M_{2}^{j}$, with j=H,V. However, to discern the correlation between spatial mode and polarization, one must first choose a specific spatial mode to examine: either $\psi_{01}(𝐫)$ or $\psi_{10}(𝐫)$ from partition 1. This is not a measurement but rather a unitary operation^3^ (by contrast, combining the projections in the Hilbert sub-space with a measurement as in previous examples, the evolution is always non-unitary, as for any projective measurements). The projection onto one of the axes in partition 1 determines the axis onto which the projection in partition 2 occurs, thus predetermining the measurement outcome on 2 before any measurement is performed: a unitary operation on 1 selecting $\psi_{01}(𝐫)$ implies $M_{2}^{H}$.

Similarly, if our aim is to resolve the type of transversal mode in the measurement, we will assign excitation to partition 1, where potential measurement outcomes are denoted $M_{1}^{j}$, with j=01,10. The projection onto one of the axes in partition 2 determines the axis onto which the projection in partition 1 occurs: a unitary operation on 2 selecting H implies $M_{1}^{01}$ prior to any measurement. Note that only one measurement on one of the partitions is possible in this case. We will delve into this case further in the next two sections.

Another property to consider, besides non-separability, is non-locality, which one would attribute to (I) and (IIa), but not to (IIb) and not, in general, to the entire set. It is worth noting that in cases (I), (IIa) and (IIb), two separate independent measurements can be performed.

From the discussion and examples above, it is evident that there are inherently different questions one needs to distinguish when seeking to assess the nature of correlations:

- Quantum-classical demarcation. What is a proper verification tool here?
- Is entanglement entirely about the non-separability of states, regardless of the physical character of the mathematical partition of a vector space?
- Is entanglement about the non-local character of correlations? What does ‘non-local’ mean? What are the appropriate means to verify it?
- When employing a non-separable state in certain protocols, where does the advantage come from?

## 3. Measurements to reveal quantumness

In this section, we address the first two previous points. We begin by emphasizing that the Bell inequality provides answers to very specific questions (as will be addressed in the next section) and requires extreme caution when applied to verify the quantumness of correlations [22]. Therefore, we take here a measurement-based, operational approach that has common points with earlier work of [16,17] and recent contributions by [22,23]. The approach of [16,17] is based on observable induced choice of Hilbert space partitioning as already discussed in §2. The most recent work of Khrennikov & Basieva [23] brings the idea of observable induced partitioning [17] even further and aims to decouple the notion of entanglement from the tensor product structure and associated non-separability. Their work emphasizes the role of the measurement process even more: Khrennikov and Basieva develop the probabilistic formalization of Schrödinger’s concept of entanglement without reference to any complex vector spaces. Entanglement is defined as dependence of observables A and B; relevant constraints are derived based on the quantum conditional probability calculus. In our approach and the approaches referred to above, choice of observables, operational access to degrees of freedom, and hence a measurement process as such, step forward as pivotal tools to assess quantum entanglement presence.

In assessing quantumness, a system cannot be considered separately from the measurement process. The key lies in the interaction between a quantum system and a measurement apparatus, with quantum mechanics providing predictions for the corresponding outcomes. Additionally, the experimental context, including the details of the apparatus used, is crucial, as it directly influences the observation process.

To assess a quantum property, one has to be sensitive to the projection imposed by a quantum measurement. This cannot be achieved by a single measurement alone; rather, one needs to perform a first measurement to impose the projection and then a second measurement to detect the effect of the projection. This is precisely what occurs in the case of quantum entanglement. A projective measurement is performed on one part of the non-separable state, A. Subsequently, a second projective measurement is performed on the other part, B, revealing the effect of the first measurement on the global state. Overall, quantum correlations inherent to entangled states are manifested through the statistically correlated results of subsequent projective measurements. Mathematically, in the partitioning of the vector space, both mode-functions A and B in the non-separable state are eigenstates of projectors, with their eigenvalues (say, 0 and 1) describing the possible outcomes of the projective measurements, denoted as $M_{A}^{0,1}$ and $M_{B}^{0,1}$, respectively, one on each of the partitions (see figure 2):


(3.1) $$\begin{array}{ccc} & |\Psi \rangle \propto |0\rangle_{A}|1\rangle_{B}+|1\rangle_{A}|0\rangle_{B}. & \end{array}$$

We see that the correlations are revealed between two statistical measurement outcomes, $C\{M_{A}^{j},M_{B}^{j}\}$.

In the case of classical entanglement, the vector space is partitioned in a fundamentally different manner (as discussed in §2b). There is only one excitation present, which can be assigned to either of the partitions spanned by available mode functions. Consider the same prototypical example as in equation (2.8):


(3.2) $$\begin{array}{ccc} & |\Psi \rangle \propto |\psi_{01}(𝐫)\rangle_{A}|H\rangle_{B}-|\psi_{10}(𝐫)\rangle_{A}|V\rangle_{B}. & \end{array}$$

For concreteness, let us assign the excitation to the polarization mode function, making the available measurement that of polarization. Then, the projective measurement will only be performed on the B-part of the non-separable functions, resulting in the projective measurement outcomes $M_{B}^{H,V}$. The projective measurement on sub-system A is replaced by some sort of mode selection, basis choice, or filter setting, and is a unitary operation (see figure 3).

To facilitate comparison with equation (3.1), let us further reformulate equations (2.8) and (3.2), highlighting the different nature of kets. The unitary operation, which distinguishes between two orthogonal basis states encoded via θ and $\theta +\frac{\pi }{2}$, is denoted $F_{\theta },F_{\theta +\frac{\pi }{2}}$. This is a filtering operation corresponding to the choice of basis, or mode, or another unitary ‘sorter’ operation. Assigning 0 and 1 to the measurement outcomes in the {H,V} basis, we obtain:


(3.3) $$\begin{array}{ccc} & |\Psi \rangle \propto |F_{\theta }\rangle_{A}|0\rangle_{B}-|F_{\theta +\frac{\pi }{2}}\rangle_{A}|1\rangle_{B}. & \end{array}$$

Compared with equation (3.1), we clearly see that there is no statistical correlation between two measurement outcomes here. Instead, we have a deterministic projective measurement outcome $M^{j}$, given the choice of basis. There is a deterministic correlation between the ‘filter’ setting k=θ,θ+π/2 and the measurement result, $C\{k,M^{j}\}$.

Entanglement *is* about non-separability, but the nature of non-separable functions is decisive. However, the key point is not whether the partitioning of the vector space, A,B, is related to spatially separated particles or to two degrees of freedom of the same particle. What is important is whether non-separable functions lead to statistically correlated measurement outcomes due to the effect of two subsequent quantum measurements, one on each sub-system (quantum entanglement, violation of realism), or whether mathematical non-separability leads to a single measurement outcome conditioned on some basis choice (classical entanglement, realism observed).

## 4. Locality and non-locality of non-separable states

In discussions comparing classical and quantum entanglement, distinctions are often made using characterizations such as ‘local’ and ‘non-local.’ But what do they mean? This question has sparked a controversial dispute that is still ongoing [6,22]. Is entanglement primarily about non-locality in the sense that an action on one part of a global system seems to have an immediate effect on the other part of the system? Or does ‘non-local’ simply mean that the system is distributed in space?

In the early 2000s, the discussion of the former aspect (is entanglement primarily about non-locality) has already led to a counterintuitive conclusion that entanglement and non-locality are different resources. As an example, a thorough investigation of incommensurability between entanglement and non-locality is presented in [24]. We return to this question a bit later in this section in the context of the measurement-based approach taken.

Regarding the latter, we have already shown in §2 that the existence of two spatially separated objects is not a necessary prerequisite for entanglement. The former is more subtle. The principle of locality states that any interaction propagates with finite velocity, and it takes time for a change in one degree of freedom to affect the other degree of freedom. On the surface, any state involving mathematically non-separable functions (equally describing either spatially separated systems or the internal degrees of freedom of one and the same system) should violate this principle of locality. Therefore, the concept of ‘spooky action at a distance’ stands as a characteristic feature of entanglement from the early Einstein–Podolsky–Rosen debates in 1935 onwards. Examples of the violation of Bell-type inequalities with classically entangled light seem to confirm this [25,26]. This leaves anyone who attributes the term ‘entanglement’ to a system that is indisputably in the quantum domain quite uncomfortable. What, then, is the decisive point that would help reliably demarcate classical correlations from genuinely quantum phenomena?

Already in this discussion of locality and non-locality, an important point emerges that enables the demarcation. First of all, recall that the violation of the Bell inequality means that quantum mechanics is not a *local realistic theory*. It is not just about locality, although we often use the term ‘non-locality’ in connection with the Bell inequality. This raises the question: Does quantum mechanics violate the locality principle, the principle of reality, or both together? The discussion is still ongoing, especially when different interpretations of quantum mechanics are debated. However, note that the violation of local-realistic assumptions occurs in a non-separable state only if a measurement is performed on one of the subsystems.

As strongly emphasized in Khrennikov [22], ‘the main deviation of classical light models from quantum theory is not only in the states but also in the description of measurement procedures’. Khrennikov argues strongly against using quantum non-locality to differentiate between quantum and classical correlations, attributing the crucial role to measurement procedures [22], which aligns well with our arguments. This ‘measurement-based’ approach provides a key to interpreting the violation of local realism. Importantly, the conventional view is that quantum theory is local, yet the assumption of realism is incorrect. This argument is quite straightforward. The principle of locality stipulates that interactions propagate with finite velocity. However, when the measurement outcome on A determines the outcome on B in non-separable states, it actually happens not due to the interaction between these two subsystems. Instead, a system in a non-separable state is in a superposition of two (in bi-partite systems) possibilities: For the state of equation (3.1), the system is simultaneously in the state $|0\rangle_{A}|1\rangle_{B}$ and $|1\rangle_{A}|0\rangle_{B}$, it is not in a definite, ‘pre-existing’ state. The distinct state emerges only upon measurement and is ‘decided’ at random in the process of measurement with a given probability.

This clearly demonstrates a violation of the principle of reality, which posits that the properties of objects are inherent and exist independently of our observations. This once again underscores the importance of the quantum measurement as a fundamental aspect of the quantum-classical boundary. The manifestation of a specific ‘reality’ during the measurement process is the source of correlations in case of the quantum entanglement. The first projective measurement, performed on A, collapses the global state in one of the possibilities, at random, say $|0\rangle_{A}|1\rangle_{B}$, leading to random ‘0’ at A. The effect of this first measurement is verified by the second measurement, which reveals the state of the second subsystem to be ‘1’, confirming statistical correlation (figure 2).

In classical entanglement, as elucidated above, a unitary process of selecting a particular basis or mode function replaces the measurement for certain operations. This process, unlike a measurement, is unitary (at least in principle), and we refer to it as ‘filtering’ or ‘sorting’. Due to the unitary nature of the operation, there is no reduction of the wave function. Hence, the mathematical non-separability in the framework of classical theory (e.g. in classical optics involving vector light fields) cannot result in any non-local effects. In general, understanding the distinction between measurement and certain unitary processes, which are sometimes confusingly interpreted as measurements, provides insight into the controversial usage of the term entanglement.

It is very tempting to exploit the analogy between wave-like properties in quantum mechanics and all the wave phenomena in classical optics (wave equation, superposition, interference, etc.). After all, in Erwin Schrödinger’s early works, quantum mechanics went under the name wave mechanics [27]. This viewpoint has been widely represented in the community, strongly highlighted in the works of Eberly *et al.* [7,8]. This analogy has also been extended to demonstrations of violations of Bell inequalities. Simulations of Bell inequalities are discussed in a number of papers, for example [25,26].

Let us depart for the moment from any specific experimental details on how it has been implemented, but let us focus on the principal idea of an experimental setting used to construct the inequality [12,13]. The statistical analysis required begins with performing *two* independent measurements on two parts of the Hilbert space, these measurements being parameterized by some parameter θ. For example, in the case of polarization entanglement of equation (2.2), the measurement process will include a polarizer, set in front of a detector and rotated at a certain angle θ. In other words, the measurement will include a detector and a ‘filter’ set for θ, as discussed previously. The inequality is constructed by combining probabilities of measurement outcomes obtained at detector 1 plus filter θ and detector 2 plus filter $\theta^{\prime }$ for particular values of θ and $\theta^{\prime }$ (for details, we refer the interested reader to [12,13]).

**Figure 4.**
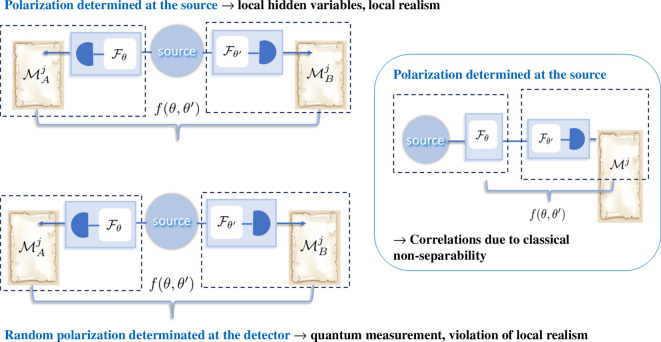
Principle experimental schemes for construction of Bell inequality for verification of polarization correlations $f(\theta ,\theta^{\prime })$. Parameterization by $\theta ,\theta^{\prime }$ happens either as a part of the measurement process, or as a part of the state preparation process, as indicated by dashed boxes. Left (top): in case of LHV assumption, the polarization is predetermined at the source, and two measurements are performed parameterized by $\theta ,\theta^{\prime }$. Left (bottom): in case of quantum entanglement, the polarization is not defined at the source and is determined in the measurement process, at the detector, when two measurements are performed, parameterized by $\theta ,\theta^{\prime }$. Right: in case of classical non-separability, light at the source is prepared in a certain polarization state, parameterized by θ. A single, parameterized by $\theta^{\prime }$ measurement is performed.

Consider now three relevant cases of polarization correlations $f(\theta ,\theta^{\prime })$: determined by local hidden variables (LHV) stemming from quantum entanglement, originating from classical non-separability. The three respective schemes are depicted in figure 4 with the example of polarization measurements. The key difference can be easily understood if we compare this figure with figures 2 and 3. The verification of the local realism assumption has been derived by Bell by examining statistical correlations of the results of *two* independent measurements on two partitions of the Hilbert space (left part of figure 4). One can simulate this test using classical non-separability, but it will not yield the same statistical analysis as performed by Bell; instead, it exploits deterministic correlations between state preparation and a single measurement to emulate Bell-like correlations (right part of figure 4).

## 5. Conclusions

Quantum and classical optics constitute an amazing field for exploring the interplay between the particle and wave nature of light [14]. The richness of wave phenomena goes beyond usual applications, allowing for the emulation of quantum features, as seen in classical entanglement, and even serving as a platform for analogue gravity experiments [28]. Despite the beauty of both quantum and classical theories of light, one should not be tempted to move the quantum-classical boundary entirely on the grounds of the presence of wave phenomena, or existence of non-separable vector spaces or the possibility of enhancing metrology protocols. Quantum mechanics is first and foremost about the interaction between a system and a measurement apparatus. Not solely the properties of the system taken on its own, nor so much the microscopic nature of the system are decisive, but the entirety of the system and the measurement process.

Assessing the quantum nature of a non-separable state requires consideration of its behaviour in a measurement process. In simple, operational language, the quantum nature of non-separable states manifests in *statistical correlations* between the outcomes of *two measurements* performed on two partitions of the corresponding Hilbert space. This distinctive feature transcends the details of state preparation taken by themselves and whether we are dealing with single photons or intense light beams.

The scheme depicted at the bottom left part of figure 4 can be utilized not only to test local realism for polarization entanglement of single photons, but also for continuous variable polarization entanglement [29]. Such non-separable states reveal quantum correlations in the measurement of Stokes operators, quantum variables with a continuous spectrum, the counterparts of classical Stokes parameters [30]. These states can be generated using intense light beams that may appear classical.

The question arises: If classical non-separability is entirely a classical phenomenon and not entanglement in its true sense, then where does the advantage demonstrated in metrology, the possibility to simulate not only Bell-like non-locality, but also a number of other quantum information protocols, come from? This is not the subject of this paper, and we will only briefly comment on it. In most cases, the advantage comes merely from the increased parameter space. One can conceptualize it as the size and structure of the Hilbert space, as seen in quantum cryptographic protocols based on structured light [31], or as a form of classical parallelism, as observed in polarization metrology [5,32].

In the same vein, this increased parameter space has been used in the pervasive field of indefinite causal order, where one can think of using one degree of freedom as control for an operation that is acting on another degree of freedom. Incorporating this control in particular tasks leads to advantages in computation [33], communication [34] and metrology [35,36], many of which have been experimentally realized [37–41].

We propose an operational distinction to clearly identify quantum entanglement and emphasize that two measurements are required for this identification. The projective quantum measurement processes by themselves remain a mystery: trying to find, where in the chain of events the projection happens is a moving target, and the ‘speed’ at which the projection happens seems to be solely determined by the response time of the detector.

## Data Availability

This article has no additional data.

## Footnotes

^1^
Not so much thinking about different degrees of freedom and their nature.

^2^
In this example we view the quantum harmonic oscillator as a discrete-variable (DV) system due to its discrete energy levels. Alternatively, one can view it as a continuous-variable (CV) system by looking at position and momentum, which requires a different framework. Note that a light mode in quantum optics can be represented as such a CV quantum harmonic oscillator [14], and in example IIa, the two output modes can be viewed as such two coupled harmonic oscillators.

^3^
A sorter or filter introduces a certain projection in the corresponding vector space, resembling quantum measurement. However, in a sorter, the state is projected and the complementary projection is in principle still available, such as in a polarizing beam splitter in optics. This unitary operation can be reverted if both output beams are used. In a quantum measurement one is necessarily ‘loosing’ information which is in principle there before the measurement. The quantum measurement projects the state of the system onto an eigenstate of the operator describing the measurement. Often a unitary sorting or filtering operation is followed by a non-unitary measurement leading to some kind of readout. The readout ’causes’ the collapse or projection of the state of the system to just one possibility. With sorting only, without any readout, all possibilities would still be open. (Note, that if you were to look only at the readout in one of the output ports of a sorter, what you see is independent of what is done at the other sorter outputs. This, by the way, is the reason why entanglement does not allow one to transfer information faster than the speed of light.
